# Regulation of Antigen Export to the Cytosol During Cross-Presentation

**DOI:** 10.3389/fimmu.2019.00041

**Published:** 2019-01-28

**Authors:** Marine Gros, Sebastian Amigorena

**Affiliations:** INSERM U932, Institut Curie, Paris, France

**Keywords:** dendritic cells, cross-presentation, cytosolic antigen export, ERAD, endosomal leakage

## Abstract

Cross-priming refers to the induction of primary cytotoxic CD8^+^ T cell responses to antigens that are not expressed in antigen presenting cells (APCs) responsible for T cell priming. Cross-priming is achieved through cross-presentation of exogenous antigens derived from tumors, extracellular pathogens or infected neighboring cells on Major Histocompatibility Complex (MHC) class I molecules. Despite extensive research efforts to understand the intracellular pathways involved in antigen cross-presentation, certain critical steps remain elusive and controversial. Here we review recent advances on antigen cross-presentation, focusing on the mechanisms involved in antigen export to the cytosol, a crucial step of this pathway.

## Introduction

Dendritic cells (DCs) play a central role in immune homeostasis by linking innate sensing to adaptive immune responses. After sampling antigens in peripheral tissues, DCs mature and migrate to lymph nodes, where they initiate adaptive immune responses by presenting processed antigens in the context of Major Histocompatibility Complex (MHC) molecules to T cells. For a long time, the generally accepted paradigm supposed that exogenous antigens were exclusively presented *via* MHC-II molecules to CD4^+^ T cells, while endogenous cytosolic antigens, derived from self or foreign proteins, were loaded on MHC-I, thereby leading to naïve cytotoxic CD8^+^ T cell activation. Yet, this simple assumption failed to explain how cytotoxic immune responses could be mounted against pathogens that do not readily infect DCs. This apparent contradiction was resolved by the discovery of cross-presentation, a process enabling the delivery of exogenous antigens to the MHC-I pathway for cross-priming CD8^+^ cytotoxic T cell responses (1, 2). Since its first description over forty years ago, our understanding of the sequence of events governing antigen cross-priming has extensively increased, leading to the description of two main pathways of antigen cross-presentation, referred to as “vacuolar” and “cytosolic.” While the requirement for cross-presentation in the initiation of anti-tumor immune responses is now well established (3–7), its control and the precise intracellular routes involved remain incompletely understood and, for some parts, controversial.

Here, we review the most recent advances in the analysis of antigen cross-presentation in mouse (unless stated otherwise), with a particular emphasis on the advances in understanding of antigen export to the cytosol, a crucial, yet debated, step of the cytosolic pathway.

## Pathways for Antigen Cross-Presentation

In 1976, seminal work by M. Bevan showed that exogenous antigens could be presented on MHC-I molecules and prime cytotoxic immune responses, thereby unearthing a novel antigen presentation pathway that he called cross-priming (1, 2). However, the molecular mechanisms underlying cross-priming and “cross-presentation” remained elusive until the early nineties. At that time, several lines of evidence reported that cross-presentation of bacterial antigens [i.e., the 257-264 H-2K^b^-restricted epitope of ovalbumin (OVA) fused to *E. coli* Crl protein] was resistant to proteasome inhibitors (8) (suggesting lysosomal processing of the corresponding peptides), unaffected by brefeldin A (BFA) treatment (8–10) [arguing against a critical role for endoplasmic reticulum (ER)-Golgi transport] and most of the time, occurred independently from TAP, the transporter mediating peptide import into the ER (8, 11). These observations led to the first description of the “vacuolar pathway.” After internalization, antigens remain confined in intracellular compartments, where they undergo lysosomal degradation, a process largely dependent on cathepsin S activity (12), and followed by loading onto post-Golgi MHC-I molecules.

Simultaneous studies with particulate, non-bacterial antigens (i.e., bead-bound OVA), showed that TAP1 deficiency in macrophages, as well as BFA treatment, abolished their ability to cross-present exogenous antigens, thereby suggesting that antigen-derived peptides must be transferred from the cytosol to the ER to bind newly synthesized MHC-I molecules (13). Additionally, cross-presentation was disrupted by proteasome inhibitors (13–16), consistent with a model in which antigens are delivered into the cytosol before proteasomal degradation and peptide import into the ER. This pathway, later termed the “cytosolic pathway,” implies the export of antigens from endocytic compartments to the cytosol. The first experimental evidence of this crucial step was provided by the use of gelonin, a membrane-impermeant toxin that inactivates ribosomes when transferred to the cytosol. Macrophages phagocytosing gelonin-coated beads displayed reduced protein synthesis, indicating export of bead-bound gelonin to the cytosol (13, 14).

The aforementioned pivotal studies used mouse macrophages as models of antigen-presenting cells (APCs). It later became clear that DCs, rather than macrophages, cross-present antigens and cross-prime cytotoxic immune responses efficiently (17, 18), by means of different properties of their phagocytic pathway, including lower degradation capacity (19). When considering DCs, these cells represent a series of ontogenically and functionally diverse populations. In mice, two main resident DC subsets are found in the spleen and lymph nodes, namely Batf3-dependent CD8^+^ XCR1^+^ DCs (DC1s) and IRF4-dependent CD8^−^ CD11b^+^ DCs (DC2s) [reviewed in (20)]. At steady state, DC1s cross-present cell-associated antigens more efficiently than their DC2 counterparts, a capacity first attributed to their increased ability to capture this type of antigen (21, 22). Later experiments showed that higher cross-presentation efficacy in mouse DC1s is intrinsic and unrelated to the route of antigen uptake (23, 24), thus contrasting with the FcγR-dependent optimization of cross-presentation observed in human DC1s (25). In mouse, surface receptors, including Clec9A/DNGR-1 (26–29) or mannose receptor (MR) (30), were proposed to preferentially deliver antigens to the cross-presentation pathway, most likely through delaying delivery of their cargoes to late endosomal and lysosomal degradative compartments. DC1s also bear specialized endocytic properties that reduce/delay acidification and degradation of endocytic cargo (19, 31).

Consistent with these *in vitro* observations, mice deficient for DC1s (5), or displaying cross-presentation-defective DCs (4, 6), fail to mount cytotoxic immune responses against tumors and to control tumor development, even after treatment with checkpoint blockers. Although DC1s are best suited for cross-presentation both *in vitro* and *in vivo*, DC2s' ability to cross-present is increased by targeting antigens to DC2 specific receptors, such as FcγR (32) or DCIR2, in a stimulatory context (33), thus suggesting that both DC1 and DC2 are capable of cross-presenting antigens depending on the conditions.

The relative contributions of the cytosolic and vacuolar pathways to *in vivo* cross-presentation and cross-priming remain unclear. TAP dependency can potentially affect both pathways, as it impairs the exit of MHC-I molecules from the ER (34–37). Whether critical players in cross-presentation, such as Sec22b (4, 38, 39) or Rab43 (40), which are both required for effective cross-priming, are selectively involved in one or both pathways is unknown. The best available evidence for the cytosolic pathway being predominant in cross-priming comes from a study using mice defective for the immunoproteasome subunit LMP7. These mice show impaired cross-priming for an immunoproteasome-dependent H-Y epitope, supporting a critical role for proteasome-dependent processing, and therefore, for the cytosolic pathway *in vivo* (41). Since delivery of internalized antigens to the cytosol is very ineffective in most cell types, DCs might have developed specialized pathways to link these two subcellular compartments.

## Biological Parameters Influencing Antigen Export to the Cytosol

### Nature of Cytosolic Export-Competent Cells

By using gelonin activity or cytosolic fluorescence quantification as readouts, initial studies showed that inflammatory (14) or activated (16) mouse macrophages displayed a measurable ability to export bead-conjugated (14) or soluble (16) cargo into the cytosol. Further work revealed that soluble or complexed antigens also get access to the cytosol in steady-state bone-marrow derived DCs (BMDCs) or in a DC cell line, without prior activation (17, 18). Moreover, antigen export to the cytosol is more efficient in DCs than in macrophages, as illustrated by subcellular fractionation and subsequent western blotting (18). To assess whether DC subsets differ in their capacity to perform such transfer, Lin et al. developed a cytochrome c-based assay relying on the selective apoptosis of cells exporting exogenous cytochrome c into the cytosol (42). Only a fraction of DC1s showed susceptibility to cytochrome c-induced apoptosis, indicating a functional specialization for endosome to cytosol transport in these cells (42, 43). Notably, this cytochrome c-sensitive DC1 population strictly corresponds to the cohort of efficient cross-presenters, whereas cytochrome c-resistant DC1s cross-present antigens inefficiently and share other functional features with DC2s (42).

### Nature of Antigens Exported to the Cytosol

Early microscopy observations showed that fluorescent (i.e., dextrans) or soluble (i.e., enzymatically active horseradish peroxidase: HRP) antigens gained access to the cytosol in DCs (17, 18). While 3–40 K dextrans are rapidly relocated to the cytosol, higher molecular mass dextrans (500–2,000 K) remain vacuolar (18), suggesting that antigen export to the cytosol is size-selective (18, 44). Particulate antigens, which are more efficiently cross-presented than soluble ones (14), often form large aggregates and therefore require dissociation before their translocation to the cytosol. Indeed, inhibition of vacuolar acidification abolishes the disaggregation of immune complexes and their subsequent cytosolic export (18), thus pointing to a crucial role of slightly acidic endo/phagosomal pH in this process. While some degree of degradation might favor antigen export to the cytosol due to the size-restriction of transported antigens (18), high proteolytic activity, favored by acidic pH, could destroy MHC-I-binding epitopes. In this regard, regulation of endocytic pH is of crucial importance. In DCs' endocytic compartments, incomplete assembly of v-ATPase proton pump together with Rab27a-dependent recruitment of NOX2 jointly lead to active alkalinization of luminal pH (19, 45), thereby preserving antigens from detrimental excessive degradation (46).

### Export to the Cytosol and DC Activation

Aside from putative intrinsic properties of DC1s, extrinsic signals, such as Toll-Like Receptor stimulation, influence antigen export to the cytosol. Indeed, short (3–5 h) lipopolysaccharide (LPS) stimulation of BMDCs increases the proportion of cells displaying exogenous HRP in their cytosol (47). A possible explanation for the observed LPS-mediated increase in antigen export may reside in the requirement for TRIF in this process (48). Until recently, absence of quantitative reliable antigen export assays based on endotoxin-free reagents impeded detailed analysis of the role of DC activation in antigen transport to the cytosol. Recently published export assays should overcome this limitation (49).

### Kinetics of Antigen Export to the Cytosol in DCs

Kinetics studies showed that HRP appeared in BMDC cytosol only 15 min after internalization (17). Rapid egress suggests that antigens are exported from early endosomes (50), as supported by microscopy experiments (51) or by mathematical modeling (52). The latter predicts that 20 min after internalization, cytosolic export of yeast-derived antigen competes with degradation associated with maturation of the endocytic compartment. Thus, only a tiny fraction of, at least, non-complexed antigens released after this time point might contribute to cross-presentation. Cytosolic translocation of HRP immune complexes appears after 60 min, and reaches a plateau after 6 h (18). Similar findings were reported for cytosolic egress of antigens associated to beads (53, 54). Additionally, these two studies provided compelling evidence that ER-mediated delivery of MHC-I loading machinery to the phagosome rendered this compartment competent for cross-presentation (55) following TAP-mediated import of cytosolic peptides (53, 54). While the relative contributions of ER and plasma membrane to the formation of cross-presentation-competent phagosomes remain debated (39, 56), Houde et al. postulated that an ER transporter, Sec61, might be involved in the translocation of antigens from the phagosomal lumen to the cytosol (53). This hypothesis was later experimentally supported by several studies detailed in the next section.

## Molecular Mechanisms of Antigen Export to the Cytosol

### ERAD Transporter-Dependent Hypothesis

**Figure 1:**
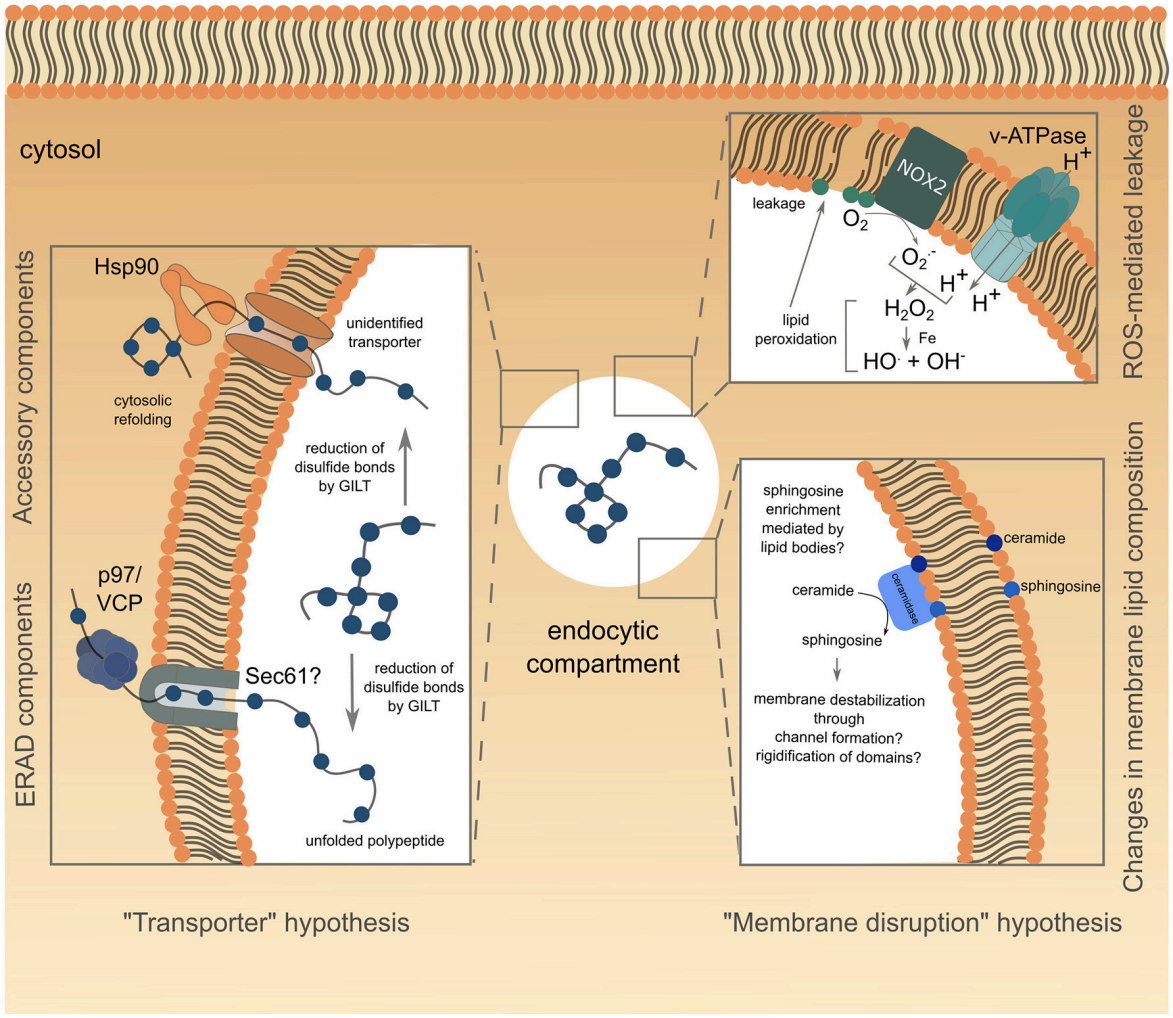
Schematic representation of the current understanding of antigen export to the cytosol during cross-presentation. The transporter hypothesis is depicted on the left side of the figure, with complexes involved grouped according to known (for ERAD) or supposed (Hsp90 with unknown transporter) interactions. The alternative membrane disruption hypothesis is depicted on the right side of the figure. The ROS-mediated leakage part has been confirmed experimentally, while modification of endo/phagosome lipid composition, suggested by biophysical studies, is speculative and lacks functional relevance in antigen export to the cytosol.

Existence of a transporter mediating antigen export to the cytosol naturally imposes conformational constraints on the translocated antigen. Indeed, antigens are unlikely to be transported in their native structure, considering the narrow diameter of known transporter pores, and are therefore expected to undergo an unfolding step before translocation. Supporting this idea, fixed OVA is less efficiently translocated into the cytosol than structurally flexible one (57). Moreover, during unfolding, reduction of disulfide bonds by GILT, a phagolysosomal thiol reductase constitutively expressed in APCs, is essential for cytosolic export of viral disulfide-rich antigens and subsequent cross-presentation (58) (Figure 1, left panel).

Although the requirement for protein unfolding suggests that antigens gain access to the cytosol through a transporter, the nature of the channel mediating this process remains controversial. Studies attempting to answer this question reported interactions between unfolded OVA and members of the ER-associated degradation (ERAD) machinery in ER-associated compartments (59), consistent with previous findings (53, 54). This observation led to the hypothesis that the ERAD machinery, mediating retro-translocation of misfolded proteins from the ER lumen to the cytosol, potentially through the trimeric Sec61 channel, could also operate from endocytic compartments during cross-presentation. The first functional insights into ERAD contributions to antigen export to the cytosol, came from studies using exotoxin A (ExoA), a bacterial toxin binding to the cytosolic N-terminal domain of Sec61α, and resulting in channel closure (60, 61). ExoA treatment reversed the ICP47-mediated inhibition of TAP, the latter resulting from the translocation of exogenously delivered ICP47 to the cytosol and its subsequent interaction with the cytosolic side of TAP (62). This finding, associated with the observed decrease in OVA cross-presentation following ExoA treatment (62, 63) or siRNA-mediated silencing of Sec61 (48, 59), strongly pointed to Sec61 being the channel controlling antigen export to the cytosol (Figure 1, left panel). In line with this hypothesis, the expression of the Sec61α, β and γ subunits is increased in DC1s, as compared to DC2s, correlating with their specific cross-presenting ability (64).

However, it has been extremely difficult to address the precise contribution of Sec61 in antigen cross-presentation and retrotranslocation from endo/phagosomes, as this channel also mediates co-translational import of proteins, including MHC-I, into the ER. To shed some light on this issue, Zehner et al. used a intrabody-based approach aiming to retain Sec61 in the ER and thereby prevent its recruitment to endocytic compartments (48). Expression of the anti-Sec61 intrabody in BMDCs impairs antigen export to the cytosol and OVA cross-presentation, consistent with a role for Sec61 outside the ER, possibly in endosomes. Still, the involvement of Sec61 itself in ERAD-dependent retrotranslocation remains unclear and fraught with technical issues [reviewed in (65)]. Additionally, recent work has shown that sustained inhibition of Sec61 with a specific toxin, mycolactone, has no effect on antigen export to the cytosol, and indirectly reduces OVA cross-presentation through downregulation of other players in the pathway, including MHC-I (66). While Sec61 involvement in cytosolic antigen translocation needs further clarification, other ERAD components, such as Hrd1 and Derlin-1, might be alternative candidates.

Hrd1, an ER-resident ubiquitin ligase tagging ERAD substrates, exhibits six transmembrane domains, which is enough to form a channel (67, 68). siRNA-mediated depletion of Hrd1 in DCs results in decreased antigen export to the cytosol and cross-presentation, as well as impaired MHC-II presentation (48). These alterations in antigen presentation pathways might be caused by Hrd1 silencing-mediated ER stress and therefore require further investigation. On the other hand, the protease Derlin-1 (Der1), comprising four transmembrane domains, cannot form a channel but could possibly function as an accessory subunit of the export channel (69) by trapping ERAD substrates and rerouting them for cytosolic degradation (70). Yet, antigen cross-presentation is not perturbed by Der1 silencing in both murine BMDCs (48) and human monocyte-derived DCs (71), thus excluding a putative role for Der1 in antigen export to the cytosol.

To date, the best evidence available suggests that ERAD might control antigen transfer to the cytosol through the activity of the AAA ATPase p97. P97 forms an hexameric ring and is thought to provide the energy necessary for passage of proteins through the retrotranslocating channel (72). Exogenous addition of p97 to isolated phagosomes loaded with luciferase leads to luciferase release, whereas addition of a dominant negative version of p97 fails to do so (62), suggesting a role for the ATPase in antigen translocation from phagosomes (Figure 1, left panel). Along the same lines, human and mouse DCs silenced for p97 (59, 71) or expressing a dominant negative form of p97 (62), display impaired cross-presentation of MelanA and OVA antigens, respectively, whereas p97 overexpression enhances this pathway (73). P97 is recruited to endosomes following mannose receptor (MR)-poly-ubiquitination. This post-translational modification proves to be crucial for antigen export to the cytosol and OVA cross-presentation as expression of a mono-ubiquitinated form of the MR is sufficient to reduce both processes (73). Of note, MR poly-ubiquitination is triggered by OVA binding to the receptor, and is negatively regulated by the ESCRT (Endosomal Sorting Complex Required for Transport)-I protein TSG-101 (73).

Several studies investigating the role of p97 in antigen export to the cytosol used the luciferase enzyme to monitor this intracellular event (62, 74). Following unfolding in endocytic compartments and subsequent translocation into the cytosol, luciferase would need to be refolded to exert its functionality, a process likely mediated by the chaperone Hsp90. Indeed, cytosolic refolding of exogenous unfolded luciferase is compromised in Hsp90β-silenced human DCs or in DCs treated with the Hsp90 inhibitor radicicol (74). Furthermore, Hsp90α deficiency not only inhibits cross-presentation in mouse BMDCs, but also decreases cytosolic translocation of OVA, therefore implying that Hsp90 itself could mediate antigen transport to the cytosol (43, 57). Additionally, Hsp90 could protect the exported antigens from premature cytosolic degradation, before Hsp70-mediated targeting to the proteasome (57) (Figure 1, left panel).

The “transporter hypothesis” has, so far, garnered the most experimental support, as the main conduit for antigen export. However, it still raises important questions. Given the high degree of substrate selectivity during ERAD [reviewed in (65)], the use of a unique transporter translocating a wide variety of antigens seems unlikely. Moreover, this hypothesis fails to explain how large, non-protein molecules, such as dextrans, can be transferred to the cytosol in absence of ubiquitination, the latter being a pre-requisite for ERAD-mediated translocation. Altogether, these observations do not exclude a role for ERAD in antigen export to the cytosol, but rather suggest the contribution of additional mechanisms.

### Alternative Hypothesis: Rupture of the Antigen-Containing Compartment

The first descriptions of the cytosolic pathway for cross-presentation supposed that antigens could escape endocytic compartments through membrane rupture. This hypothesis, at that time termed “indigestion model,” relies on the observation that large particles are more efficiently cross-presented than small ones, and could thus be responsible for phagosomal overload, leading to membrane disruption and efficient antigen leakage to the cytosol (14). Despite intensive use of this pathway for cytosolic delivery of antibodies or bioactive proteins conjugated with endosomolytic peptides (75–77), evidence of its contribution to cross-presentation were lacking, until recently.

#### ROS, Lipid Peroxidation, and Membrane Rupture

A recent study showed that following LPS stimulation, VAMP8-dependent NOX2 recruitment to BMDC endosomes resulted in Reactive Oxygen Species (ROS) production and subsequent endosomal lipid peroxidation (78, 79). This alteration of lipid structure disrupts endosomal membrane integrity, leading to antigen escape to the cytosol and OVA cross-presentation (78) (Figure 1, upper right panel). Interestingly, ROS production in endocytic compartments is intrinsically linked to cells' cross-presenting ability. Indeed, DCs show sustained and stronger ROS production than macrophages (19), the latter subset increasing phagosomal ROS production, as well as cross-presentation, only after activation (17, 19). Moreover, ROS generation is higher in DC1 than in DC2 phagosomes, thereby correlating with the enhanced ability of DC1s to cross-present antigens (31). Biophysical studies provided mechanistic insights into lipid peroxidation-dependent membrane rupture. Oxidized lipid-rich artificial bilayers show higher water permeability (80), as well as increased membrane curvature, associated with micellization and membrane destabilization (81).

#### Changes in Endolysosomal Membrane Lipid Composition

Aside from lipid peroxidation, enrichment in ceramide, and to a greater extent in sphingosine, also triggers membrane permeability to solutes (82). While some studies proposed that sphingosine-based lipids could form large channels in membranes through an “all or none” mechanism (83), others suggested that sphingolipids actually promote membrane permeabilization by a graded process involving rigidification of membrane domains and subsequent creation of local structural defects (82, 84) (Figure 1, lower right panel). Sphingosine synthesis results from ceramide deacetylation by two ceramidases, encoded by the Asah1 and Asah2 genes, and respectively functioning at acid or neutral pH. Notably, the expression of both enzymes is higher in DC1s than in DC2s (immgen.org), suggesting that ceramide conversion into membrane-disrupting sphingosine could be increased in DC1 endocytic compartments. This DC1-specific enrichment in sphingosine could possibly be mediated by lipid bodies, which have also been proposed to destabilize some ER or phagosomal membrane domains during their formation, thereby causing leakage of the content of these compartments (85). Along the same line, BMDCs deficient for Igtp, a GTPase controlling accumulation of lipid bodies, show a selective defect for cross-presentation (86). Moreover, intracellular accumulation of lipid droplets correlates with cross-presentation efficiency, as DC1s display significantly higher amounts of these organelles than DC2s (86). However, pharmacological interference with lipid body formation fails to influence antigen export to the cytosol in the context of saponin adjuvant-based cross-presentation (87). Thus, the precise role of lipid bodies in cross-presentation and cytosolic antigen leakage remains to be specified.

#### Compensatory Mechanisms for Endocytic Membrane Rupture

Although several lines of evidence point to a contribution of endocytic membrane disruption and subsequent antigen leakage into the cytosol, this model has been repeatedly dismissed owing to its presumable lack of regulation and ensuing cell toxicity. Indeed, links between endocytic leakage and cell death were reported in different systems. Silica crystal-dependent phagosomal rupture, for example, leads to cytosolic release of intraluminal cathepsin B, which in turn activates the NLRP3 inflammasome, resulting in pyroptosis (88). Hydroxychloroquine-mediated cathepsin release from lysosomes can also trigger caspase activation and apoptosis (89), suggesting a requirement for control mechanisms to contain damaging consequences of leakage.

In this regard, the ESCRT machinery, formerly known for its key role in viral budding or cytokinetic abscission (90, 91), was recently identified as a core component of biological membrane repair following damage (92–96). A role for the ESCRT-I protein TSG101 in antigen export to the cytosol and cross-presentation has been previously suggested (73). However, increased cytosolic export observed following TSG101 silencing had been attributed to TSG101-dependent inhibition of MR poly-ubiquitination, required for cytosolic antigen translocation. Yet, considering the dispensable role of ubiquitination in antigen export to the cytosol (43) and the fact that TSG101 is also required for ESCRT-III-mediated repair of endolysosomal membranes (95, 96), defects in endocytic membrane repair, concomitant with TSG101 depletion, may have also contributed to the observed increased export phenotype (73). Yet, the possible involvement of ESCRT-III in controlling antigen export to the cytosol has not been investigated so far.

## Conclusion

Identification of several critical players in antigen cross-presentation, such as Sec22b (4), or Rab43 (40), and their subsequent validation in conditional knock-out mouse pre-clinical models established a major role for this pathway in different types of immunes responses, including anti-tumor immune responses. Yet, the way antigens gain access to the cytosol during cross-presentation is far from being entirely resolved. Export to the cytosol is not only the last event in the pathway that remains largely obscure, but it is also a rate-limiting step in the process (52, 97). Identifying the molecular mechanism involved will certainly provide relevant targets to manipulate antigen cross-presentation for vaccination and immunotherapy purposes.

## Author Contributions

MG and SA designed, prepared and wrote the manuscript.

### Conflict of Interest Statement

The authors declare that the research was conducted in the absence of any commercial or financial relationships that could be construed as a potential conflict of interest.
